# The Transcultural Community Resilience Scale: Psychometric Properties and Multinational Validity in the Context of the COVID-19 Pandemic

**DOI:** 10.3389/fpsyg.2021.713477

**Published:** 2021-08-19

**Authors:** Jude Mary Cénat, Rose Darly Dalexis, Daniel Derivois, Martine Hébert, Saba Hajizadeh, Cyrille Kossigan Kokou-Kpolou, Mireille Guerrier, Cécile Rousseau

**Affiliations:** ^1^School of Psychology, University of Ottawa, Ottawa, ON, Canada; ^2^Interdisciplinary School of Health Sciences, University of Ottawa, Ottawa, ON, Canada; ^3^Université Bourgogne Franche Comté, Dijon, France; ^4^Department of Sexology, Université du Québec à Montréal (UQAM), Montréal, QC, Canada; ^5^Division of Social and Transcultural Psychiatry, McGill University, Montréal, QC, Canada

**Keywords:** Transcultural Community Resilience Scale psychometric properties, depression, multinational validation, multinational sample, multilingual sample

## Abstract

Few instruments assess community resilience. In the midst of the COVID-19 pandemic, the capacity of communities to support resilience of members deserves to be assessed to develop programs for improving mental health of affected populations. This article presents the development of the Transcultural-Community Resilience Scale (T-CRS), its underlying factorial structure and transcultural validity with a multilingual (English, French, Creole, Kinyarwanda), multinational (DR Congo, Haiti, Rwanda, Togo) and multicultural sample affected by this pandemic. A sample of 1,267 participants (40.9% women) were recruited in the four countries: DRC (*n* = 626, 43.4% women), Haiti (*n* = 225, 42.0% women), Rwanda (*n* = 174, 40.5% women), and Togo (*n* = 242, 33.2% women), with a mean age of 32 (SD = 10.1). They completed measures assessing individual resilience, depression and the T-CRS. Exploratory and confirmatory Factor Analyses, Cronbach alpha, coefficient H and the McDonald's Omega, and bivariate regression were used to estimate the underlying components of the T-CRS, its internal consistency and concurrent validity. Parallel factorial analysis and confirmatory factor analysis results revealed an excellent fit 3-factor structure. Internal consistency coefficients varied between 0.82 and 0.95. The T-CRS showed a good construct validity with a positive association with individual resilience and negative association with depression score. Developed with a collaborative approach involving researchers, practitioners, and clients/patients, the T-CRS and its three factors (community strengths and support, community trust and faith, and community values) demonstrated excellent psychometric properties for assessing community resilience among adults during the COVID-19 pandemic.

## Introduction

Over the past four decades, more and more research has been devoted to the study of resilience (Rutter, 1985; Wagnild and Young, 1993; Bonanno et al., 2007; Cénat and Derivois, 2014; Gagnon and Stewart, 2014; Masten, 2014; Cénat et al., 2018). These studies aimed to better understand the relationship between the adversities and traumas experienced by children, adolescents and adults, their psychopathological consequences and their ability to cope, rebuild and rebound (Hoge et al., 2007; Fossion et al., 2013; Gagnon and Stewart, 2014; Pritzker and Minter, 2014; Cénat et al., 2015a, 2020; Kokou-Kpolou et al., 2020). Studies on resilience have been conducted on human behavior, in medical sciences, in health psychology, as well as with survivors of different forms of trauma: interpersonal trauma (child maltreatment, sexual assault, partner violence, etc.), natural disasters (earthquakes, floods, etc.), man-made disasters (wars, torture, etc.), among others (Betancourt and Khan, 2008; Martinez-Torteya et al., 2009; Zraly and Nyirazinyoye, 2010; Tsai et al., 2012; Cénat and Derivois, 2014; Cénat et al., 2015a, 2019, 2021a; McCanlies et al., 2018). Although there is no agreement on the definition of resilience, current studies tend to define it as a process that allows a person or group to rebuild and bounce back after experiencing adversity or one or multiple traumas (Rutter, 1985, 1987; Ungar and Liebenberg, 2011; Masten, 2014). Viewing it through a more comprehensive lens than early definitions, we defined resilience, in previous studies, as a psycho-socio-eco-process (Cénat et al., 2013, 2015a; Cénat and Derivois, 2014). That is, it is a personal process arising from interactions between risk and protective factors, as well as the individual, social, ecological, cultural and community resources at the disposal of an individual or group who has faced adversity and trauma (Cénat et al., 2013, 2015a; Cénat and Derivois, 2014; Cénat, 2015).

To understand those personal processes, several measures have been developed to assess the resilience of individual people in different settings and following different events (Wagnild and Young, 1993; Connor and Davidson, 2003; Friborg et al., 2003; Hjemdal et al., 2011; Ungar and Liebenberg, 2011; Windle et al., 2011). Among these measures, we can cite the most widely used scales, such as the Connor-Davidson Resilience scale (CD-RISC) (Connor and Davidson, 2003), the Resilience Scale (RS) (Wagnild and Young, 1993), Resilience Scale for Adults (RSA) (Friborg et al., 2003), and the Brief Resilience Scale (BRS) (Smith et al., 2008), which are the four with the best psychometric properties according to a review of resilience instruments (Windle et al., 2011). The collective dimension of resilience is now attracting more attention, raising important definitional and measurement challenges. This paper presents the development of a collective resilience instrument, validated in four Low-and middle-income countries (LMIC) during the COVID-19 pandemic.

### Defining and Measuring Community Resilience

Although the majority of studies agree on the role of the social environment and of community resources on the development of resilience, few tools have been developed to assess community resilience (Horton and Wallander, 2001; Ozbay et al., 2007; Derivois et al., 2020). This scarcity of tools stems from the fact that, to date, definitions of community resilience have diverged, as have the different theoretical approaches used to conceptualize it (Norris et al., 2008; Castleden et al., 2011; Leykin et al., 2013; Patel et al., 2017). The most widely used definition in the literature is that of Norris et al. (2008, p. 131) who defined community resilience as “*A process linking a set of networked adaptive capacities to a positive trajectory of functioning and adaptation in constituent populations after a disturbance*.” However, as shown by a recent systematic review, no consensus has been reached regarding the definition of community resilience (Patel et al., 2017). On the contrary, data from 80 studies included in this review revealed significant discrepancies in the way community resilience is defined. Nevertheless, the authors noted three types of definitions: “(1) ‘process' definitions (i.e., *an ongoing process of change and adaptation*); (2) ‘absence of adverse effect' definitions (i.e., *an ability to maintain stable functioning*), and (3) ‘range of attributes' definitions (i.e., *a broad collection of response-related ab*ilities)” (Patel et al., 2017, p. 6). They also found nine core constituent elements of community resilience: *local knowledge, community networks and relationships, communication, health, governance and leadership, resources, economic investment, preparedness, and mental outlook*. However, the literature review we conducted in order to develop the Transcultural Community Resilience Scale (T-CRS) enabled us to highlight two principal ways of defining community resilience. The first is characterized by the processes that govern a community's ability to adapt and return to a functional communal life following a collective trauma (Castleden et al., 2011; Kulig et al., 2013; Lyons et al., 2016). The second is the capacity of a community to avail itself of resources that can facilitate the resilience of its members (Norris et al., 2008; Lovell et al., 2014; Lindberg and Swearingen, 2020). While the first definition makes it difficult to measure community resilience, the second makes it easier to be operationalized. To date, the vast majority of measures that evaluate community resilience are based on the first definition and aim to describe the strengths of a community, but are based on individual responses, which are then combined in an attempt to operationalize the resilience of that community. Among them, there is the Communities Advancing Resilience Toolkit (CART) that evaluates community resilience across four components: Connection and Caring, Resources, Transformative Potential, and Disaster Management (Pfefferbaum et al., 2013). Next, the Conjoint Community Resiliency Assessment Measure (CCRAM), is a 21-item and five-factor questionnaire designed to *"describe a community's ability to deal with crises or disruptions”* (Leykin et al., 2013, p. 313). However, measures according to the second definition are relevant because they may be much more operational than measures that assess individuals to better understand a collective variable. This lack of measures to assess the capacity of communities to provide their members with the resources necessary to become resilient motivated the development of this scale called The Transcultural Community Resilience Scale (T-CRS).

### Development of the Transcultural Community Resilience Scale (T-CRS)

The development of the T-CRS stands on the definition of community resilience as the capacity of communities to avail themselves of resources to facilitate the resilience of their members (Norris et al., 2008; Patel et al., 2017). The necessity of the development of this scale stems from the strong links consistently observed in studies between social support, quality of family ties, membership to positive community groups, and individual resilience (Horton and Wallander, 2001; Ozbay et al., 2007; Ungar et al., 2013; Cénat et al., 2018; Derivois et al., 2020). To develop this tool, we first conducted a literature review in order to identify the principal elements emerging from publications on the capacity and resources of communities to foster the resilience of their members. In total, six main factors (Community support, Community competence, Community coping strategies, Community trust and faith, Community strengths, Sense of belonging: Community Bonds, Roots, and Commitments), as well as 41 different elements were identified.

Based on the factors and elements found in this preliminary stage, the Vulnerability, Trauma, Resilience and Culture Research Laboratory team at the University of Ottawa developed 88 different items. We then took a collaborative and transcultural approach, inviting different researchers and practitioners from different countries and cultures to review the different items. As such, the 88 items were submitted to 28 researchers and practitioners who have worked and published on the subject of individual and community resilience, but come from different fields (psychology, medicine, social work, neurosciences, sociology, anthropology, and education) and from 17 different countries (Canada, USA, France, Germany, Democratic Republic of the Congo, Haiti, Togo, Rwanda, Nigeria, Russia, Brazil, Chile, Senegal, Benin, Iran, China, Pakistan). Their task consisted of answering the following questions for each item: (1) whether or not to include it (yes or no); (2) why they were in support of or against its inclusion; (3) propose corrections, criticisms and comments for each item, if necessary; and finally, (4) whether additional items should be added.

This process resulted in the elimination of 49 items and the addition of four additional ones. Thus, after this careful analysis of the many changes, 39 items were retained. These items were then submitted to 13 other researchers who have worked on community resilience following different forms of trauma, and have at least three publications in the field. They were asked to answer the same questions as the first group of researchers. This process resulted in the elimination of 11 items and the creation of one new item. A total of 29 items were therefore retained with the 6 factors listed above (Community support: 4 items, Community competence: 4 items, Community coping strategies: 6 items, Community trust and faith: 5 items; Community strengths: 6 items, Sense of belonging: Community Bonds, Roots, and Commitments: 4 items).

Finally, we conducted 12 cognitive interviews with adult trauma survivors who had undergone or were undergoing psychotherapy in order to identify critical information about the subjective comprehension underlying each response. This allowed us to carry out an initial analysis of the content validity of the questionnaire, meaning, to test whether each item was representative of the construct it is supposed to measure.

### Objectives

This article first aims to describe the development of the Transcultural Community Resilience Scale (T-CRS). Second, it aims to study the underlying factorial structure of the T-CRS, test its transcultural validity and investigate its psychometric properties in a multinational and multilingual sample from Haiti, DR Congo, Rwanda and Togo during the COVID-19 pandemic.

## Methods

### Study Design and Participants

A total of 1,267 participants (40.9% women) were recruited through social media including Facebook, Twitter and WhatsApp, and also *via* telephone from March to May 2020, during the COVID-19 pandemic. Participants were recruited from four countries: DRC (*n* = 626, 43.4% women), Haiti (*n* = 225, 42.0% women), Rwanda (*n* = 174, 40.5% women), and Togo (*n* = 242, 33.2% women), with a mean age of 32 (*SD* = 10.1). All participants signed an informed electronic consent form or gave their vocal consent. The ethics committees of the University of Ottawa (H-04-20-5712) and the Institut National pour la Recherche Biomedicale (National Institute for Biomedical Research) of DRC approved the study protocol More details on the study protocole can found in Cénat et al. (2021b).

This research was conducted while these four countries were implementing containment measures due to the COVID-19 pandemic. As of October 12, these countries have been affected by the pandemic to varying degrees: The Democratic Republic of the Congo (DRC; 10,868), Haiti (8,882 confirmed cases), Rwanda (4,905), and Togo (1,949), with death totals related to COVID-19, of 276, 230, 32, and 49 deaths, respectively (John Hopkins University, 2021).

### Measures

Participants completed the questionnaire in the language of their choice: English, French, Creole, or Kinyarwanda. All participants completed a socio-demographic questionnaire.

### The Transcultural Community Resilience Scale (T-CRS)

The Transcultural Community Resilience Scale (T-CRS) contained 29 items during the administration of the questionnaire. Respondents are asked to indicate their level of agreement with each one, using a Likert scale ranging from 1 (strongly disagree) to 5 (strongly agree). Developed in English, the O-CRS has been translated into French, Creole and Kinyarwanda using the World Health Organization standards for translation-retranslation with local language and mental health experts.

### Individual Resilience

The 2-item shortened version of the Connor-Davidson Resilience Scale (CD-RISC2) (Connor and Davidson, 2003; Vaishnavi et al., 2007) was used to assess individual resilience. This measure includes the items 1 and 8 of the full length 25-item scale, completed on a Likert scale ranging from 0 to 4 (not true at all, rarely true, sometimes true, often true, and true nearly all of the time (item 1: “Able to adapt to change,” and item 8: “Tend to bounce back after illness or hardship”). It is a widely used measure with good internal consistency in different cultures. Cronbach's alpha was 0.72 in our sample.

### Depression

We used the depression subscale of the Hopkins Symptom Checklist (HSCL) to assess depressive symptoms (Winokur et al., 1984). The depression subscale of the HSCL contains 15 items completed with a 4-point scale from “Not at all” (1), “A little” (2), “Quite a bit” (3), “Extremely” (4). The HSCL was used in different cultures and showed strong psychometric properties (Lee et al., 2008). In our sample, Cronbach's alpha was 0.91.

### Statistical Analysis

The Mean, Standard Deviation, Skewness, and Kurtosis of each item of the Transcultural Community Resilience Scale were computed and examined. Exploratory Factor Analysis (EFA) and Confirmatory Factor Analysis (CFA) methods were used to estimate the underlying components of Community Resilience among the population. The combined sample was randomly split in half considering each country; sample 1 (*n* = 615) was used for EFA and sample 2 (*n* = 652) for CFA.

**Figure 1:**
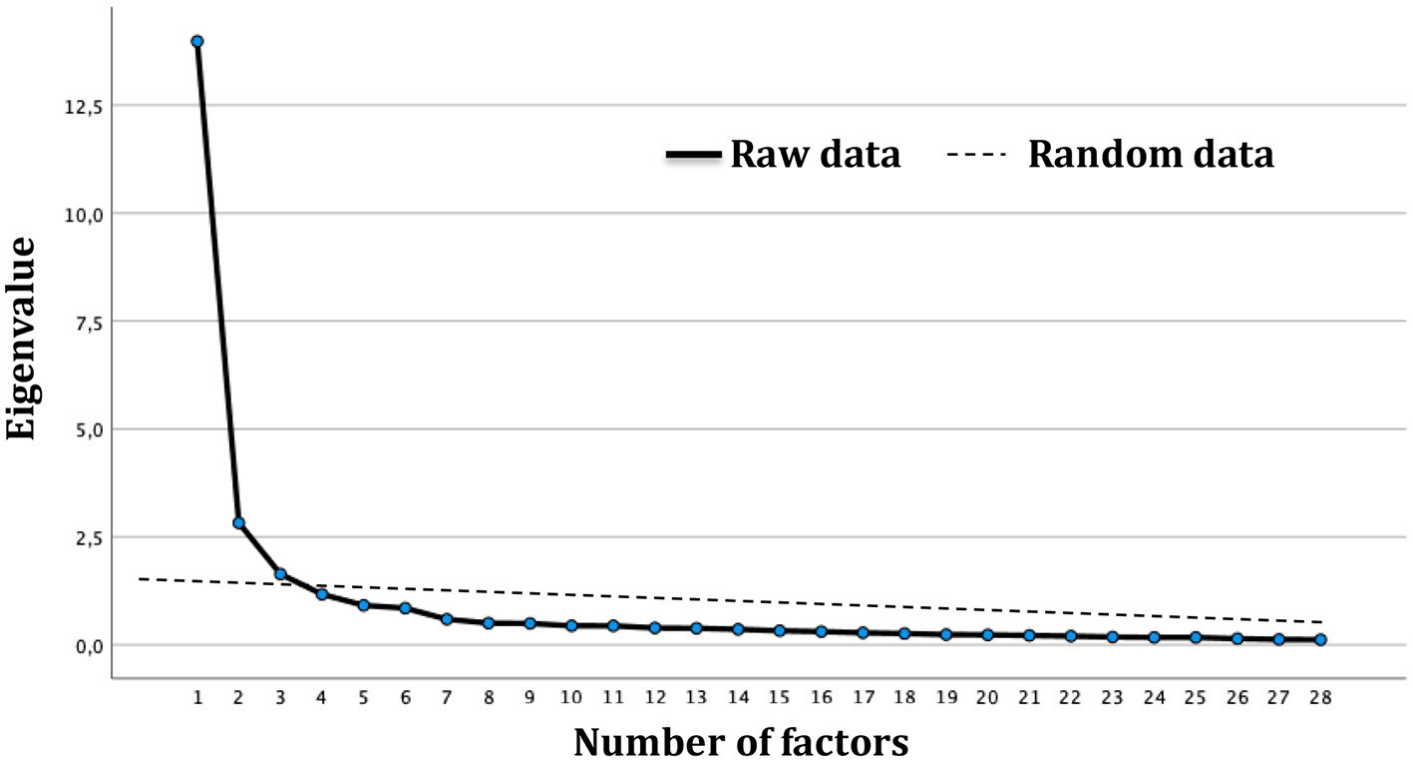
Scree plot from the principal axis analysis and parallel analysis conducted on Resilience Scale in subsample 1.

Prior to the extraction of the factors, the Kaiser-Meyer-Olkin (KMO) test and Bartlett's Test of Sphericity were conducted to assess the suitability of the data to factor analysis. A KMO value >0.8 and significant specificity test indicate good sampling adequacy (Bartlett, 1950; Kaiser, 1970; Tabachnick and Fidell, 2007). EFA was performed using oblique rotation which produces factor structures that are correlated. Apart from considering the Kaiser's criterion (eigenvalue > 1) and the Scree plot, the number of factors retained in the analysis was also probed using the cumulative percentage of variance (see Figure 1). Items with factor loading >0.40 were retained (Kaiser, 1960; Horn, 1965; Cattell, 1966).

The model with the factor solution that best fit the above-mentioned criteria and interpretability was then carried to the CFA using the other half of our sample. The CFA method was performed to assay and validate the number of factors retained from EFA. EFA was performed using Maximum-Likelihood; the models were validated using Root Mean Square Error of Approximation (RMSEA), Adjusted Goodness of Fit (AGFI), Incremental Fit Index (IFI), and Comparative Fit Index (CFI. Values of RMSEA <0.06, CFI and AGFI >0.9 indicate a good fit of the data (Bollen, 1989; Hu and Bentler, 1999). In view of the increasing critical comments regarding Cronbach's alpha as internal consistency measure, we have also decided to calculate the coefficient H and the McDonald's Omega (McNeish, 2018). Pearson bivariate correlation analyses were also performed to study the concurrent validity of the T-CRS using scores of depression and individual resilience. Analyses were conducted using SPSS Version 27 and Statistica Version 12.

## Results

### Descriptive Analyses

Descriptive Statistics (Mean, SD, Skewness, and Kurtosis) of Transcultural Community Resilience Scale items are summarized in Table 1. All items have values of Skewness ranging between −2 and 2, and Kurtosis between −7 and 7 which suggest acceptable overall normality (Byrne and van de Vijver, 2010).

**Table 1:** Distribution of Mean, standard deviation (SD), Skewness, and Kurtosis of the community resilience items (*N* = 615; subsample 1).

	**Items**	**Mean**	**SD**	**Skewness**	**Kurtosis**
1	If anything was to happen to me, I know I could count on my community	2.96	1.35	0.05	−1.12
2	In the event of an extreme situation (natural disaster, war, etc.), I know that I can count on my community to face the event and move forward	3.04	1.34	−0.02	−1.12
3	When I go through hard times, there are people in my community I can talk with	3.22	1.34	−0.20	−1.11
4	The relationships I maintain in my community help me cope with problems that happen to me or that may happen	3.16	1.30	−0.17	−1.04
5	One of my strengths in the face of adversity is knowing that I can count on one or many people from my community	3.11	1.33	−0.12	−1.10
6	The members of my community know they can count on me when problems arise	3.33	1.33	−0.33	−1.03
7	I am willing to help the members of my community who face difficulties	3.54	1.33	−0.55	−0.82
8	I get involved in my community's activities	3.37	1.30	−0.39	−0.91
9	My cultural traditions and spiritual and/or religious and/or my values help me cope with difficulties	3.37	1.36	−0.34	−1.07
10	My community's activities help me create bonds with people	3.24	1.36	−0.22	−1.15
11	My community helps me adapt in the event of changes or difficulties	3.02	1.32	−0.03	−1.05
12	Being able to count on my community in the event of difficulties is very reassuring to me	3.06	1.31	−0.08	−1.06
13	In my community, we always find a way to laugh and distract ourselves, even in difficult times	3.18	1.32	−0.21	−1.06
14	In my community, there is at least one person who can help me find concrete solutions when I face difficulties	3.26	1.34	−0.25	−1.08
15	When I go through difficult times, there are institutions in my community and/or my city that can help me	2.79	1.33	0.15	−1.09
16	If I were to get sick, I know that I could turn to the health care institutions in my area to have the care necessary	2.98	1.38	0.04	−1.19
17	I trust the health care staff in my area to provide me with adequate care	2.97	1.32	0.00	−1.08
18	I have trust in the social services of my community	2.76	1.31	0.15	−1.07
19	I have enough information to know which institutions to turn to in the event of difficulties	3.01	1.36	−0.05	−1.17
21	In my community, there are important traditions of mutual support	2.91	1.32	0.08	−1.07
22	My community makes efforts to integrate all its members and to make them stronger	2.91	1.32	0.04	−1.07
23	My community enables its different members to build strong bonds	2.99	1.32	−0.07	−1.08
24	Mutual support is one of the values in my community	3.1	1.33	−0.11	−1.09
25	In my community, sharing is a very important value	3.15	1.34	−0.17	−1.12
26	I feel proud to be a member of my community	3.28	1.32	−0.25	−1.05
27	I share the values of my community	3.24	1.31	−0.21	−1.04
28	Participating in my community's activities is important to me	3.29	1.32	−0.28	−1.04
29	I am attached to my community and to its values	3.24	1.34	−0.23	−1.09

### Exploratory and Confirmatory Factorial Analyses

KMO value of 0.95 and significant Bartlett Sphericity test (χ^2^ = 11,590.14; *p* < 0.001) showed good fit of the data to the analysis. Based on eigenvalue >1 and Scree plot, a total of three (3) factors were retained from EFA (see Figure 1). Among the 29 items, 1 item (*My community is strong enough to cope with extreme situations*) was removed from the 3-factor solution for cross-loading; Table 2 summarizes the factor loading of the including items. Factor 1, which was named Community strengths and support, encompasses 14 items; 5 items were grouped under Factor 2 named Community trust and faith; and, lastly, the last dimension identified as Community values, includes 9 items.

**Table 2:** Exploratory factorial analyses (factor loadings and explained variance) and internal consistency analyses (*N* = 615; subsample 1).

**Items**	**Factors**		
	**1**	**2**	**3**
If anything was to happen to me, I know I could count on my community	0.75	0.18	0.09
In the event of an extreme situation (natural disaster, war, etc.), I know that I can count on my community to face the event and move forward	0.72	0.19	0.14
When I go through hard times, there are people in my community I can talk with	0.73	0.16	0.14
The relationships I maintain in my community help me cope with problems that happen to me or that may happen	0.74	0.27	0.08
One of my strengths in the face of adversity is knowing that I can count on one or many people from my community	0.76	0.19	0.10
The members of my community know they can count on me when problems arise	0.66	0.17	0.14
I am willing to help the members of my community who face difficulties	0.64	0.20	0.12
I get involved in my community's activities	0.60	0.27	0.21
My cultural traditions and spiritual and/or religious and/or my values help me cope with difficulties	0.62	0.24	0.12
My community's activities help me create bonds with people	0.63	0.28	0.22
My community helps me adapt in the event of changes or difficulties	0.65	0.28	0.35
Being able to count on my community in the event of difficulties is very reassuring to me	0.61	0.31	0.35
In my community, we always find a way to laugh and distract ourselves, even in difficult times	0.55	0.37	0.31
In my community, there is at least one person who can help me find concrete solutions when I face difficulties	0.49	0.30	0.32
When I go through difficult times, there are institutions in my community and/or my city that can help me	0.28	0.32	0.57
If I were to get sick, I know that I could turn to the health care institutions in my area to have the care necessary	0.23	0.31	0.66
I trust the health care staff in my area to provide me with adequate care	0.15	0.23	0.80
I have trust in the social services of my community	0.11	0.25	0.74
I have enough information to know which institutions to turn to in the event of difficulties	0.16	0.28	0.68
In my community, there are important traditions of mutual support	0.25	0.63	0.25
My community makes efforts to integrate all its members and to make them stronger	0.22	0.72	0.34
My community enables its different members to build strong bonds	0.24	0.76	0.28
Mutual support is one of the values in my community	0.25	0.77	0.25
In my community, sharing is a very important value	0.25	0.77	0.23
I feel proud to be a member of my community	0.30	0.74	0.24
I share the values of my community	0.28	0.75	0.22
Participating in my community's activities is important to me	0.27	0.72	0.24
I am attached to my community and to its values	0.29	0.74	0.24
Explained Variance	24.50	21.83	13.00
Cronbach's Alpha	0.95	0.95	0.88
McDonal's Omega	0.91	0.91	0.82
Indice H	0.92	0.92	0.83

The above-mentioned model was then submitted to the CFA using the sample 2; fit indices showed overall good fit of the data to the model: RMSEA value of 0.042, AGFI = 0.92; IFI = 0.91, and CFI = 0.96 (See Table 3). We also performed CFA for sample from each country (Haiti, DRC, Rwanda, and Togo). The results also showed excellent goodness of fit indices for each country separately, except for the Rwanda where the IFI was just below the recommended value of 0.90 (0.89).

**Table 3:** Confirmatory factorial results over countries.

	**Haiti**	**DRC**	**Rwanda**	**Togo**	**Total**
RMSEA	0.050	0.033	0.053	0.046	0.042
AGFI	0.94	0.96	0.90	0.91	0.92
IFI	0.91	0.94	0.89	0.90	0.91
CFI	0.95	0.97	0.92	0.94	0.96

### Internal Consistency and Concurrent Validity of the Transcultural Community Resilience Scale

The 28-item structure of the Transcultural Community Resilience Scale showed excellent internal consistency (respectively, Cronbach Alpha = 0.96, McDonald's Omega = 0.97, and coefficient H = 0.96). The 14 items of the Factor 1 also showed excellent internal consistency (Cronbach Alpha = 0.95, McDonald's Omega = 0.91, and coefficient H = 0.92); as well as the five items of the Factor 2 (Cronbach Alpha = 0.95, McDonald's Omega = 0.91, and coefficient H = 0.92); and the 9 items of the Factor 3 (Cronbach Alpha = 0.88, McDonald's Omega = 0.82, and coefficient H = 0.83).

We investigated the concurrent validity of the measure using Pearson bivariate correlations between T-CRS score and individual resilience and depression scores. As expected, the T-CRS total score was positively correlated with individual resilience (*r* = 0.41, *p* = 0.0001) and negatively correlated with depression (*r* = −0.26, *p* = 0.0001). Table 4 shows the same pattern for bivariate correlations among the three factors of the T-CRS and individual resilience and depression scores.

**Table 4:** Correlations of resilience and the other scales (*N* = 652; subsample 2).

	**Individual resilience**	**Depression**
O-CRS total score	0.41^***^	−0.26^***^
Community strengths and support (Factor 1)	0.49^***^	−0.29^***^
Community trust and faith (Factor 2)	0.22^***^	−0.11^**^
Community values (Factor 3)	0.37^***^	−0.25^^**^*^

** *p < 0.01,*

*** *p < 0.001*.

## Discussion

The first objective of this article was to describe the development of the Transcultural Community Resilience Scale (T-CRS). The use of a collaborative approach in developing this measure, with researchers from different countries and cultures, and from different fields (psychology, medicine, social work, neurosciences, sociology, anthropology, and education), has attempted to give it a truly transcultural character, within the very heterogeneous Afro-Caribbean geo-cultural area. The various exchanges, comments, criticisms, and additions from researchers and practitioners from North America, the Caribbean, Europe, Africa, Asia throughout the development of the T-CRS have led to the development of a measure that can, first, capture the extent of the support that people are likely to receive from their local communities, in spite of cultural differences; and second, how this support is used by individuals to cope with adversity and trauma, and to rebuild and rebound. Also, cognitive interviews with patients/clients were important to ensure that items are understandable, did not use scientific jargon, but included items that measured what they intended to measure. Ultimately, this scale was developed using a collaborative approach, bringing together researchers, practitioners and patients/clients. In addition, existing measures to assess resilience are generally designed around the definition that communities are able to return to a functional status after experiencing significant adversities (Castleden et al., 2011; Kulig et al., 2013; Lyons et al., 2016). However, these measures assessed individuals to conceptualize a collective variable. The present scale is based on an approach that assesses the ability of communities to provide the resources necessary to facilitate the resilience of its members (Norris et al., 2008; Lovell et al., 2014; Lindberg and Swearingen, 2020). It is the best way to assess how communities experience adversities and how they help their members cope and bounce back. Hence the importance of this measure which provides a much more operationalizable conceptualization of community resilience.

The second objective of this article was to investigate the underlying structures of the T-CRS in a multinational (Haiti, DR Congo, Rwanda, and Togo) and multilingual (English, French, Creole, Kinyarwanda) sample. The results of this study showed a three-factor structure, comprised of 14, 5, and 9 items, respectively. The three-factor underlying structure that emerged from this multicultural, multinational, and multilingual sample respond to the theoretical dimensions that led to the development of the T-CRS. In fact, after removing the cross-loaded item, the grouping of the 28 items was as initially anticipated. However, we had previously anticipated six factors, but three (Community support, Community competence, Community coping strategies) were grouped together to form the *Community strengths and support* subscale; the items of the *Community trust and faith* subscale remained grouped together; while the last two factors *(Community strengths, Sense of belonging: Community Bonds, Roots, and Commitments)* were grouped together and renamed *Community values*. All three dimensions fit very well with the definition that characterizes community resilience as the ability of communities to provide their members with the resources necessary to become resilient. Given the collective and collectivist nature of this characteristic, the more communities have the capacity to help their members to develop their resilience, the more resilient they (communities) are themselves.

The third objective of this study was to analyze the transcultural validity and psychometric properties of the T-CRS. First, confirmatory factor analyses showed that the underlying structure of the questionnaire was very adequate with excellent goodness of fit indices for this multicultural, multilingual, and multinational sample. Second, the results also revealed that the T-CRS has excellent overall internal consistency and the subscales have very good internal consistency with Cronbach's alpha, H and omega coefficients ranging from 0.82 to 0.95 (McNeish, 2018). The results also showed that the scale has good concurrent validity when compared to an individual resilience scale and to a measure of depression. For depression, this is consistent with what previous studies have shown, i.e., a negative correlation with resilience (Kukihara et al., 2014; Cénat et al., 2015a; Wermelinger Ávila et al., 2017). With respect to individual resilience Leykin et al. (2013) found similar results between perceived community resilience and perceived individual resilience.

### Toward a More Comprehensive Definition of Community Resilience

Given the ambiguity that exists among prevailing definitions of community resilience, the promising psychometric properties of this scale confer us the opportunity to propose a more comprehensive definition. It is based on the holistic development of the questionnaire which incorporates both what has been written to date on community resilience in the scientific literature, as well as the views of researchers, practitioners, and patients/clients. This research allows us to define community resilience as a set of processes characterized by the capacity of communities to make available the resources, support, and interactions necessary to enable individual members to cope with individual and collective trauma, to rebuild and rebound, while helping other community members to do the same. It is therefore a reciprocal and two-way process nourished by community strengths and support, community trust and faith, and community values, which provides a sense of community to individuals, enabling them to rely on other members and structures to build their resilience, while other members can also rely on their support to develop their own.

### Limitations

While this study demonstrates good psychometric properties of the Transcultural Communitiy Resilience Scale, it also has limitations. The first relates to the fact that we recruited a convenience sample. The second is associated with the fact that the sample was recruited partly over the Internet and partly by telephone. Although we made a considerable effort to recruit as many participants as possible via telephone, people in all four countries have limited access to the Internet and information technology. However, these measures have been important and have been recommended by ethics committees to prevent the spread of the COVID-19. The third limitation is related to the cross-sectional design of this study. Indeed, a longitudinal design would have allowed us to also evaluate the test-retest reliability of the T-CRS. The language issues in these countries could also have been a limitation, but we responded as best we could by making the T-CRS available in the languages of instruction and vernacular languages, when it seemed necessary. Finally, future studies should test the association between T-CRS and variables other than individual resilience and depression, as well as its moderating effect in the association between trauma/adversity and outcomes.

## Conclusions

This study showed that the Transcultural Community Resilience Scale has very good psychometric properties and multinational and multilingual validity in Afro-Caribbean populations affected by the COVID-19 pandemic. This scale allows the evaluation of the process of building resilience through the resources, supports, and connections that communities provide to individuals and that help them to cope with individual and collective adversity and trauma. It is a brief, self-administered, with subscales that have good internal consistency and can be used separately, if necessary.

This study provides a scale that can be used in both clinical and research contexts. We strongly recommend it for studies in populations facing collective trauma in order to better predict the need for resources, supports, interconnections, and symbols to help individuals build resilience (Norris and Stevens, 2007; Norris and Wind, 2009; Pfefferbaum et al., 2013; Derivois and Cénat, 2014; Cénat et al., 2015b, 2018; Lyons et al., 2016; Derivois et al., 2020). It can also be used with people who have faced adversity, and communities in a minority context (Cénat, 2020), to not only measure the resources and supports available to them in their community, but also their ability to connect and use them to cope, rebuild and rebound.

## Data Availability Statement

The raw data supporting the conclusions of this article will be made available by the authors, without undue reservation.

## Ethics Statement

The studies involving human participants were reviewed and approved by Research Ethics Board of the University of Ottawa. The patients/participants provided their written informed consent to participate in this study.

## Author Contributions

JMC, RDD, CKK-K, and MG: conceptualization. JMC, CKK-K, RDD, CR, and DD: investigation and acquisition of data. MG and JMC: software and formal analysis. JMC, MG, CKK-K, RDD, MH, DD, and CR: interpretation of data. JMC, MG, RDD, and SH: writing—original draft. CR, DD, MH, and JMC: writing—review and editing. All authors contributed to the article and approved the submitted version.

## Conflict of Interest

The authors declare that the research was conducted in the absence of any commercial or financial relationships that could be construed as a potential conflict of interest.

## Publisher's Note

All claims expressed in this article are solely those of the authors and do not necessarily represent those of their affiliated organizations, or those of the publisher, the editors and the reviewers. Any product that may be evaluated in this article, or claim that may be made by its manufacturer, is not guaranteed or endorsed by the publisher.
