# Corrigendum: The Transcultural Community Resilience Scale: Psychometric Properties and Multinational Validity in the Context of the COVID-19 Pandemic

**DOI:** 10.3389/fpsyg.2021.771706

**Published:** 2021-10-08

**Authors:** Jude Mary Cénat, Rose Darly Dalexis, Daniel Derivois, Martine Hébert, Saba Hajizadeh, Cyrille Kossigan Kokou-Kpolou, Mireille Guerrier, Cécile Rousseau

**Affiliations:** ^1^School of Psychology, University of Ottawa, Ottawa, ON, Canada; ^2^Interdisciplinary School of Health Sciences, University of Ottawa, Ottawa, ON, Canada; ^3^Université Bourgogne Franche Comté, Dijon, France; ^4^Department of Sexology, Université du Québec à Montréal (UQAM), Montréal, QC, Canada; ^5^Division of Social and Transcultural Psychiatry, McGill University, Montréal, QC, Canada

**Keywords:** transcultural community resilience scale psychometric properties, depression, multinational validation, multinational sample, multilingual sample

In the original article, there was an error in the abstract as published. There was an error in the name of the “Transcultural-Community Resilience Scale” and its abbreviation “T-CRS.”

A correction has been made to the *Abstract*. The corrected paragraph is shown below.

Few instruments assess community resilience. In the midst of the COVID-19 pandemic, the capacity of communities to support resilience of members deserves to be assessed to develop programs for improving mental health of affected populations. This article presents the development of the Transcultural-Community Resilience Scale (T-CRS), its underlying factorial structure and transcultural validity with a multilingual (English, French, Creole, Kinyarwanda), multinational (DR Congo, Haiti, Rwanda, Togo) and multicultural sample affected by this pandemic. A sample of 1,267 participants (40.9% women) were recruited in the four countries: DRC (*n* = 626, 43.4% women), Haiti (*n* = 225, 42.0% women), Rwanda (*n* = 174, 40.5% women), and Togo (*n* = 242, 33.2% women), with a mean age of 32 (SD = 10.1). They completed measures assessing individual resilience, depression and the T-CRS. Exploratory and confirmatory Factor Analyses, Cronbach alpha, coefficient H and the McDonald's Omega, and bivariate regression were used to estimate the underlying components of the T-CRS, its internal consistency and concurrent validity. Parallel factorial analysis and confirmatory factor analysis results revealed an excellent fit 3-factor structure. Internal consistency coefficients varied between 0.82 and 0.95. The T-CRS showed a good construct validity with a positive association with individual resilience and negative association with depression score. Developed with a collaborative approach involving researchers, practitioners, and clients/patients, the T-CRS and its three factors (community strengths and support, community trust and faith, and community values) demonstrated excellent psychometric properties for assessing community resilience among adults during the COVID-19 pandemic.

The authors apologize for this error and state that this does not change the scientific conclusions of the article in any way. The original article has been updated.

## Publisher's Note

All claims expressed in this article are solely those of the authors and do not necessarily represent those of their affiliated organizations, or those of the publisher, the editors and the reviewers. Any product that may be evaluated in this article, or claim that may be made by its manufacturer, is not guaranteed or endorsed by the publisher.

